# EMS non-conveyance: A safe practice to decrease ED crowding or a threat to patient safety?

**DOI:** 10.1186/s12873-021-00508-1

**Published:** 2021-10-09

**Authors:** Jani Paulin, Jouni Kurola, Mari Koivisto, Timo Iirola

**Affiliations:** 1 Department of Clinical Medicine, University of Turku and Turku University of Applied Sciences, Turku, Finland; 2 Centre for Prehospital Emergency Care, Kuopio University Hospital and University of Eastern Finland, Kuopio, Finland; 3 Department of Biostatistics, University of Turku, Turku, Finland; 4 Emergency Medical Services, Turku University Hospital and University of Turku, Turku, Finland

**Keywords:** Emergency medical service, Non-conveyance, Adverse outcome, Patient safety

## Abstract

**Background:**

The safety of the Emergency Medical Service’s (EMS’s) non-conveyance decision was evaluated by EMS re-contacts, primary health care or emergency department (ED) visits, and hospitalization within 48 h. The secondary outcome was 28-day mortality.

**Methods:**

This cohort study used prospectively collected data on non-conveyed EMS patients from three different regions in Finland between June 1 and November 30, 2018. The Adjusted International Classification of Primary Care (ICPC2) as the reason for care was compared to hospital discharge diagnoses (ICD10). Multivariable logistic regressions were used to determine factors that were independently associated with adverse outcomes. Results are presented with adjusted odds ratios (aORs) together with 95% confidence intervals (CIs). Data regarding deceased patients were reviewed by the study group.

**Results:**

Of the non-conveyed EMS patients (*n* = 11,861), 6.3% re-contacted the EMS, 8.3% attended a primary health care facility, 4.2% went to the ED, 1.6% were hospitalized, and 0.1% died 0–24 h after the EMS mission. The 0–24 h adverse event rate was higher than 24–48 h. After non-conveyance, 32 (0.3%) patients were admitted to an intensive care unit within 24 h. Primary non-urgent EMS mission (aOR 1.49; 95% CI 1.25 to 1.77), EMS arrival at night (aOR 1.82; 95% CI 1.58 to 2.09), ALS unit type vs BLS (aOR 1.43; 95% CI 1.16 to 1.77), rural area (aOR 1.74; 95% CI 1.51 to 1.99), and older patient age (aOR 1.41; 95% CI 1.20 to 1.66) were associated with subsequent primary health care visits (0–24 h).

**Conclusions:**

Four in five non-conveyed patients did not have any re-contact in follow-up period. EMS non-conveyance seems to be a relatively safe method of focusing ED resources and avoiding ED crowding.

## Background

Emergency Medical Services (EMSs) and emergency departments (EDs) have reported increased workload [1, 2], mainly due to an aging population and difficulties accessing primary care [2]. The role of the EMS has changed to include more non-critical emergency patients [3], and patients are increasingly assessed and treated at the scene by EMSs, avoiding unnecessary conveyance to EDs [4]. Reported non-conveyance rates substantially vary internationally, between 3.7 and 93.7% in the general population [5]; in Finland, the rate is approximately 40% [3, 6, 7].

The decision-making process for non-conveyance appears to be complex and multifactorial [5]. EMS care providers’ higher education level [3, 8], EMS arrival time in the evening or at night [3, 7, 9, 10], longer distance to a healthcare facility [7], rural area, younger patient age [3, 10, 11], low National Early Warning Score (NEWS2), and alcohol use increase the likelihood of non-conveyance [3]. A recent review showed that, after a non-conveyance decision, re-contact with the EMS or the ED, hospitalization and mortality rates varied a lot [5]. However, whether re-contact with EMS was for a similar reason as the initial EMS contact is unclear [5].

Assessment and triage are a central part of the EMS work process [4]. Under-triaging may put patients’ safety at risk, whereas over-triaging leads to inappropriate use of limited resources [12]. NEWS2 developed by the Royal College of Physicians is a simple, widely adopted scoring system [13]. NEWS2 may help identify patients at risk of deterioration who need to be treated and conveyed by the EMS, but whether it can be used as an indicator regarding EMS non-conveyance decisions is controversial [14, 15].

Patient safety is a priority of the EMSs. Decisions not to convey patients may represent a risk to patient safety. These safety factors are unclear in the prehospital setting [4, 16] and relevant studies are lacking [5, 17]. However, some studies indicate that EMSs are able to make accurate preliminary diagnoses [18]. In the EMS context, a great number of adverse outcomes are associated with difficulties in clinical judgement [16] and patient groups with non-specific reasons for care [19, 20]. Older age and abnormal vital signs are common predictors of adverse outcomes after non-conveyance and, therefore, pose a threat to patient safety [21]. Finally, from the patient safety perspective, little is known about non-conveyance decisions and related adverse outcomes [5, 17, 22].

The aim of this study was to identify the rate and predictors of adverse outcomes after non-conveyance by the EMSs to determine whether the current practice of non-conveyance ensures patient safety.

## Methods

### Design

This is a prospective cohort study.

### Finland’s health care system and EMS

Finland is one of the five Nordic welfare states. The health care system is financed by public funds and mainly organized by public sector. Health services are divided into primary and specialized medical care.

Organized by 21 hospital districts, EMSs are part of specialized care. In a four-tiered system, ambulance units are normally at the ALS level manned by at least one paramedic-nurse with a 4-year bachelor-level education. The other person in an ALS unit or personnel in a BLS unit can be a firefighter, an emergency medical technician (EMT) or a practical / registered nurse. A non-conveyance decision can be made supported by regional or national guidelines or by consulting a 24/7 on-call EMS or primary care physician. If EMS conveyance is needed, the target ED or other health care facility is decided upon by the EMS care provider with consultation of the EMSs or primary care physician if necessary [23].

A national dispatch authority operates with the common 112 emergency number in six regional emergency medical communication centers (EMCCs). Medical emergency calls are classified into four categories (A, B, C, and D), with A and B being urgent calls with lights and sirens. All dispatchers have completed 18 months of education, but they are usually not health care providers.

### The EMS data

**Fig. 1:**
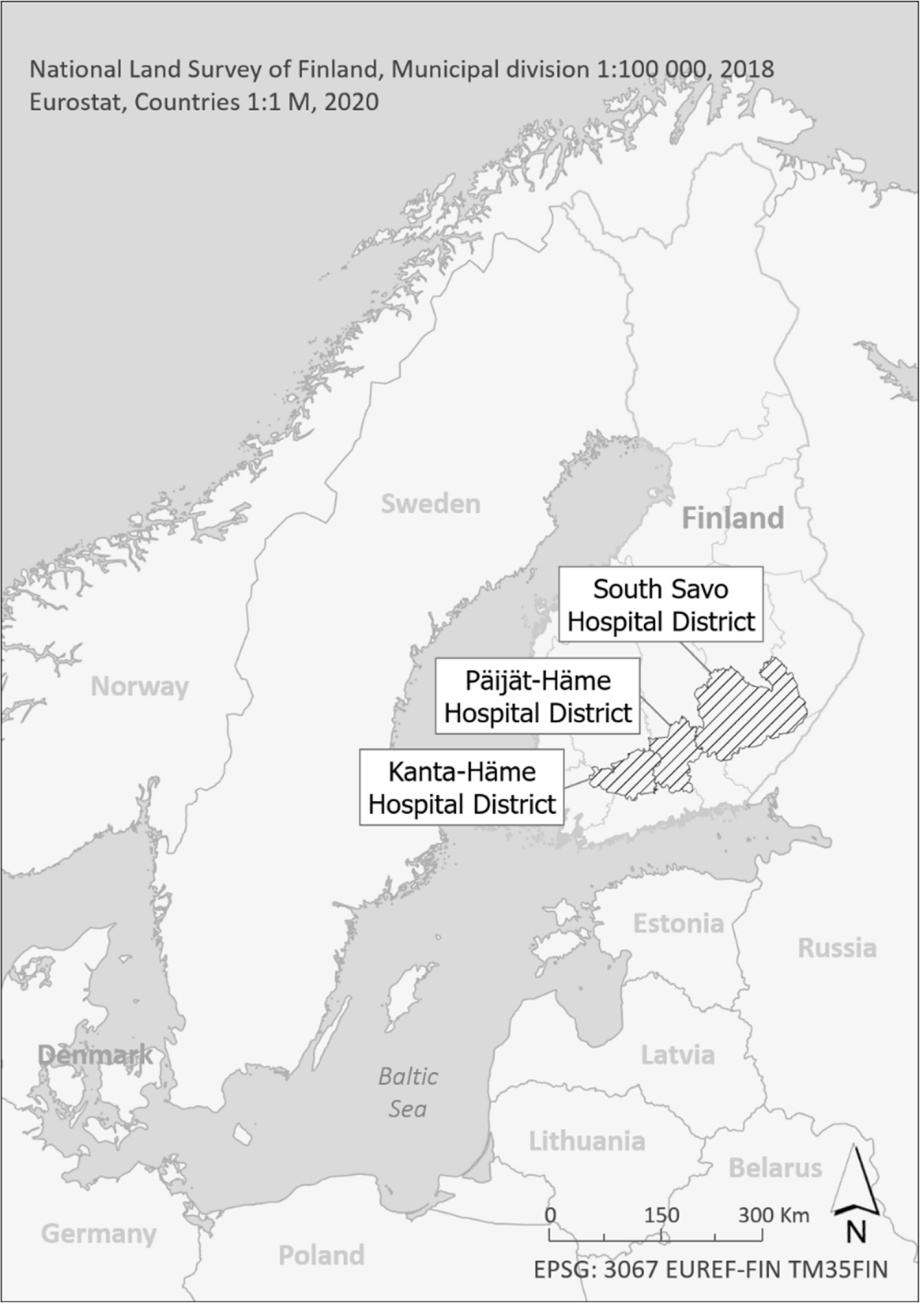
Study areas

The EMS data were collected between June 1 and November 30, 2018, from the data systems of the Finnish hospital districts of South-Savo, Kanta-Häme, and Päijät-Häme (Fig. 1). The study area, consisting of 32 municipalities and both urban and rural areas, has 482,805 inhabitants, which is 8.8% of the total Finnish population. The average population density is 26.1 people per square kilometer. The adjusted ICPC2 classification as the main reason for EMS care was taken in to use. The original ICPC2 coding is used in primary care and published by WHO [24, 25]. The adjusted classification (around one hundred ICPC2 codes) was created by the Nordic Collaboration (Benchmarking) Group for the context of prehospital emergency care [26] and it is published in the code server of the Finnish Institute for Health and Welfare [27]. The EMS care providers were trained in the use of the codes before the study period. More detailed description of the adjusted ICPC2 classification, measurement and interpretation of the NEWS2 scores, urban-rural classification, distance to health care facilities, use of alcohol, and additional data collection were described previously [3].

### Study protocol

**Fig. 2:**
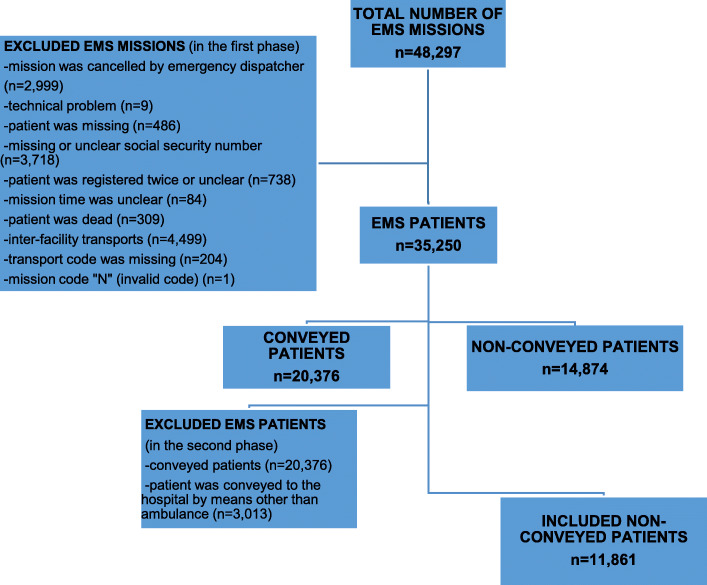
Flow chart

Non-conveyed patients who were discharged at the scene after EMS assessment and treatment were included in this analysis. Exclusion criteria are presented in Fig. 2. Included patients were identified using unique 10-digit personal identity numbers and linked to the registries described below.

The first EMS re-contact was recorded between 0 and 24 h and 24–48 h from the initial non-conveyance EMS mission. If the re-contact did not lead to conveyance, a new follow-up period was started. The main reason for care (ICPC2; preliminary diagnosis) was compared between the initial contact and the EMS re-contact, i.e., whether the re-contact was related to the initial non-conveyance mission.

In Finland, hospital districts are required to submit care notifications to registries. Therefore, registry information on visits to primary health care facilities or EDs and hospitalization is available from the Finnish Institute for Health and Welfare [28, 29]. Unscheduled visits to primary health care and EDs (0–24 h or 24–48 h) were collected. If the exact time of the visit was missing, the initial non-conveyance case was judged to have occurred first and the 0–24 h health care visit to have occurred the same day or the day after. However, in the Register of Primary Health Care Visits [28], the data also include chronic disease monitoring. The first visit was analyzed and combined with the latest non-conveyance case if there were many. Whether the patient went to hospital by ambulance or by other means was not recorded. The ICPC2 code chosen by the EMS was compared to the main discharge diagnosis (ICD10) based on ICPC2 and ICD10 mapping charts to determine whether the visit was for the same or a related complaint [25].

The Finnish Causes of Death registry data, including death certificates from Statistics Finland [30], were used to identify deceased patients. The deaths were considered for a longer time to gain deeper insight (28 days from initial non-conveyance mission). In the data, the time of death was registered only by date, not by hour. Thus, the 0–24 h mortality includes deaths that occurred the same and the following day as the initial non-conveyance case. Only unexpected deaths were analyzed; end of life patients were excluded from the analysis because they normally have formal arrangements for dying at home. The death was connected with the last non-conveyance mission. Two experienced emergency physicians (JK and TI) analyzed all cases independently; if the judgement differed, the case was discussed until consensus was reached (JP, JK, and TI). Deceased patients were evaluated as follows: 1) Was the death related to end-of-life care? 2) Did the patient refuse conveyance to the ED or primary health care facility? 3) Was the death credibly connected to the initial non-conveyance case? 4) Would the patient have benefited from conveyance to an ED or primary health care facility?

### Outcome measures

The primary outcomes were EMS re-contact, unscheduled primary health care or ED visit, and hospitalization in 0–24 and 24–48 h. The secondary outcome was 28-day mortality.

### Data analysis

Categorical variables were characterized using frequencies and percentages and continuous variables using medians and interquartile range (IQR). The age groups were defined based on the Finnish national classification provided by Statistics Finland. Distance to nearest primary health care facility or ED was classified for the purposes of the analysis.

Univariate associations between outcome variables and categorical study variables were studied using logistic regression analysis. Multiple logistic regression analysis included variables that were clinically and statistically significant after univariate analysis. The NEWS2 score is suitable only for patients > 16 years of age, and distance to the nearest primary health care facility or ED measures the same thing as rural-urban classification; therefore, these factors were excluded from the model. Moreover, a non-specific reason for care (ICPC2) as the categorical variable and hospitalization in 24–48 h and 28-day mortality as dependent variables were infrequent in the data. Thus, these analysis were not performed. Results are presented with univariate and adjusted odds ratios (ORs and aORs) together with 95% confidence intervals (CIs) and *p*-values.

All analyses included only patients who did not have missing values for variables included in the model. Statistical analyses were carried out using SAS for Windows version 9.4 (SAS Institute Inc., Cary, NC, USA), and *p* < 0.05 was considered significant.

## Results

A total of 48,297 EMS missions were identified, with 35,250 EMS missions included for the first-phase analysis. Of these patients, 42% (*n* = 14,874) were treated and discharged at the scene. Of these non-conveyed patients, 11,861 met the final inclusion criteria for this study (Fig. 2, Table 1). The reasons for non-conveyance are given in Table 2. The median age of the included patients was 67 (IQR 44–80) years, and 52.6% were females. Regarding EMS contacts during the study period, 85% (*n* = 7887) had 1 contact, 14.4% (*n* = 1334) had 2–6 contacts, and 0.6% (*n* = 60) had at least 7 contacts. Overall, 16% of the patients were under the influence of alcohol. The NEWS2 score was low, with 54.5% of patients having zero points (Table 3).

**Table 1:** Characteristics of non-conveyed patients (*N* = 11,861)

	Missing	n	%
Mission priority	5		
A		413	3.5
B		2694	22.7
C		5534	46.7
D		3215	27.1
EMS unit			
ALS		9627	81.2
BLS		2002	16.9
Community Paramedic		228	1.9
Field Supervisor		4	0.03
Doctor at scene		32	0.3
Doctor consulted by phone		5118	43.2
Day of week			
Monday		1504	12.7
Tuesday		1482	12.5
Wednesday		1578	13.3
Thursday		1635	13.8
Friday		1865	15.7
Saturday		2014	17.0
Sunday		1783	15.0
EMS arrival time	9		
08:00–20.00		6383	53.9
20:00–08:00		5469	46.1
Urban–rural classification	176		
Urban area		7198	61.6
Rural area		4487	38.4
Distance to nearest health care facility	177		
< 5 km		3871	33.1
5–20 km		3941	33.7
21–40 km		2578	22.1
> 40 km		1294	11.1
Median distance 8 km [IQR 3.0–24.6]			
Mission duration: median 52 min [IQR 39–69]

**Table 2:** Reasons for non-conveyance (*n* = 11,861)

	n	%
**Treated at scene or there was no need for conveyance.**	10,713	90.3
**Patients refused conveyance.**	736	6.2
**Patients were handed over to the police.**	306	2.6
**Patients received other help, such as homecare.**	106	0.9

**Table 3:** NEWS2 score (age > 16 years)

NEWS2 score	Clinical risk	Non-conveyed patients (*N* = 11,861, missing 536)
**Aggregate score 0–4**	Low	10,338 (91.3)
**Red score**; **Score of 3 in any individual parameter**	Low–medium	714 (6.3)
**Aggregate score 5–6**	Medium	215 (1.9)
**Aggregate score ≥ 7**	High	58 (0.5)
**Median [IQR]**		0 [0–1]

Data are given as n (%) unless otherwise noted

After the non-conveyance decision, adverse event rates were as follows: EMS re-contact 0–24 h 6.3%, 24–48 h 2.6%; primary health care facility attendance 0–24 h 8.3%, 24–48 h 2.6%; ED attendance 0–24 h 4.4%, 24–48 h 0.8%; hospitalization after ED contact 0–24 h 1.6%, 24–48 h 0.3%; and death 0–24 h 0.1%, 24–48 h 0.03%, within 28 days 1.1%. Some of these patients had multiple types of adverse events, but 4 in 5 (83.9% in 0–24 h) did not have any (Table 4). Reasons for care (ICPC-2) are presented in Table 5.

**Table 4:** Pathway analyses of adverse events in 0–24 h

	EMS	Primary health care	ED	Hospitalization	Death	n	%
Did not have any re-contact	x	x	x	x	x	9951	83.9
Primary health care attendance	x	**✓**	x	x	x	805	6.8
EMS re-contact	**✓**	x	x	x	x	478	4.0
ED attendance	x	x	**✓**	x	x	233	2.0
EMS re-contact and primary health care attendance	**✓**	**✓**	x	x	x	80	0.7
EMS re-contact and ED attendance	**✓**	x	**✓**	x	x	74	0.6
ED attendance and hospitalization	x	x	**✓**	**✓**	x	73	0.6
EMS re-contact, ED attendance and hospitalization	**✓**	x	**✓**	**✓**	x	65	0.6
Primary health care and ED attendance and hospitalization	x	**✓**	**✓**	**✓**	x	35	0.3
Primary health care and ED attendance	x	**✓**	**✓**	x	x	20	0.2
Death	x	x	x	x	**✓**	17	0.1
EMS re-contact, primary health care and ED attendance and hospitalization	**✓**	x	**✓**	**✓**	x	11	0.1
EMS re-contact and death	**✓**	x	x	x	**✓**	9	0.1
EMS re-contact, primary health care and ED attendance	**✓**	**✓**	**✓**	x	x	5	0.04
EMS re-contact, ED attendance and death	**✓**	x	**✓**	x	**✓**	3	0.03
Primary health care attendance and death	x	**✓**	x	x	**✓**	2	0.02

**Table 5:** The initial ICPC2–codes of non-conveyed patients before subsequent events

**EMS re-contacts 0–24 h (*n*** **= 652, missing 73)**					**EMS re-contacts 24–48 h (*n*** **= 262, missing 31)**				
**ICPC2**		n	%		**ICPC2**		n		%
A04	Weakness/tiredness, general	99	15.2		A04	Weakness/tiredness, general	49		18.7
P16	Acute alcohol abuse	80	12.3		A97	No disease	34		13.0
A97	No disease	78	12.0		P16	Acute alcohol abuse	28		10.7
L02	Back symptom/complaint	39	6.0		L02	Back symptom/complaint	17		6.5
R02	Shortness of breath/dyspnea	38	5.8		P29	Psychological symptom/complaint other	15		5.7
D01	Acute abdomen	28	4.3		A11	Chest pain	13		5.0
A11	Chest pain	22	3.4		R02	Shortness of breath/dyspnea	10		3.8
P29	Psychological symptom/complaint other	22	3.4		D01	Acute abdomen	8		3.1
A01	Pain general	19	2.9		N17	Vertigo/dizziness	7		2.7
N07	Convulsion/seizure	17	2.6		N01	Headache	6		2.3
**Visit to primary health care 0–24 h** **(*n*** **= 877, missing 81)**					**Visit to primary health care 24**–**48 h** **(***n* **= 263, missing 23)**				
**ICPC2**		n	%		**ICPC2**		n		%
A04	Weakness/tiredness, general	118	13.5		A04	Weakness/tiredness, general	38		14.5
L02	Back symptom/complaint	52	5.9		A97	No disease	21		8.0
D01	Acute abdomen	52	5.9		D01	Acute abdomen	18		6.8
A11	Chest pain	49	5.6		K80	Other cardiac arrhythmia	16		6.1
A97	No disease	48	5.5		L02	Back symptom/complaint	14		5.3
P16	Acute alcohol abuse	33	3.8		A11	Chest pain	12		4.6
N17	Vertigo/dizziness	32	3.7		A01	Pain general	11		4.2
A03	Fever	30	3.4		N17	Vertigo/dizziness	9		3.4
K85	High blood pressure	29	3.3		P29	Psychological symptom/complaint other	9		3.4
A01	Pain general	23	2.6		P16	Acute alcohol abuse	8		3.0
**Visit to ED 0–24 h (*n*** **= 438, missing 81)**					**Visit to ED 24**–**48 h (*n*** **= 78, missing 8)**				
**ICPC2**		n	%		**ICPC2**		n		%
A04	Weakness/tiredness, general	49	11.2		A04	Weakness/tiredness, general	11		14.1
A97	No disease	46	10.5		L02	Back symptom/complaint	8		10.3
D01	Acute abdomen	28	6.4		A97	No disease	6		7.7
L02	Back symptom/complaint	27	6.2		A11	Chest pain	6		7.7
P16	Acute alcohol abuse	25	5.7		D01	Acute abdomen	4		5.1
P29	Psychological symptom/complaint other	15	3.4		P16	Acute alcohol abuse	3		3.9
A01	Pain general	15	3.4		A03	Fever	3		3.9
L17	Foot/toe symptom/complaint	13	3.0		A06	Fainting/syncope	3		3.9
A03	Fever	12	2.7		P29	Psychological symptom/complaint other	2		2.6
A11	Chest pain	11	2.5		A92	Allergy/allergic reaction NOS	2		2.6
**Hospitalization 0–24 h (*n*** **= 155,missing 29)**					**Hospitalization 24**–**48 h (*n*** **= 26, missing 1)**				
**ICPC2**		n	%		**ICPC2**		n		%
A04	Weakness/tiredness, general	24	15.5		A04	Weakness/tiredness, general	5		17.2
A97	No disease	16	10.3		A11	Chest pain	2		6.9
L02	Back symptom/complaint	11	7.1		A97	No disease	2		6.9
P16	Acute alcohol abuse	10	6.5		A87	Complication of surgical procedure	2		6.9
L17	Foot/toe symptom/complaint	10	6.5		D01	Acute abdomen	2		6.9
D01	Acute abdomen	8	5.2		L02	Back symptom/complaint	2		6.9
A11	Chest pain	6	3.9		A06	Fainting/syncope	2		6.9
A03	Fever	6	3.9		L04	Chest symptom/complaint	2		6.9
P29	Psychological symptom/complaint other	6	3.9		A01	Pain general	1		3.5
A01	Pain general	5	3.2		K74	Ischemic chest pain	1		3.5

In the case of EMS re-attendance in 0–24 h, the re-contact was related (same ICPC-2 code) to the initial mission in 36.1% of cases, for a different reason in 50.5%, and the ICPC2 code was missing in 13.4% of missions. The corresponding figures for EMS re-attendance in 24–48 h were 29.0, 55.0, and 16.0%, respectively. Of these EMS re-contacts in 0–24 h, in 4 of 5 cases (80.4%) the mission priority was non-urgent, ending in a new non-conveyance decision in 39.7%; the corresponding figures for 24–48 h are 79.5 and 50.6%, respectively. The NEWS2 points were as follows: at 0–24 h, 50.8% had zero points and 83.3% had 0–4 points, whereas at 24–48 h 53.0% had zero points and 85.5% had 0–4 points. The median duration of visits to primary health care was 15 min and 65% of the patients were discharged from the ED within 24 h. The majority of primary health care or ED ICD10 diagnoses were not mapped to the initial EMS ICPC2 codes. Among the non-conveyed patients, 0.3% were admitted to an intensive care unit in 0–24 h (0.03% in 24–48 h) and 0.5% to high dependency units (0.1% in 24–48 h; Table 6).

**Table 6:** Characteristics of study outcomes

	0–24 h, n	0–24 h, %	24–48 h, n	24–48 h, %
**EMS re-contacts**	725	6.3	293	2.6
Mission priority				
non-urgent	583	80.4	222	75.8
urgent	142	19.6	71	24.2
ended in a non-conveyance decision	288	39.7	148	50.6
NEWS2 points				
zero	366	50.8	156	53.8
0–4	600	83.3	248	85.5
EMS re-contact association with initial non-conveyance mission				
ICPC2 code same	262	36.1	85	29.0
ICPC2 code different	366	50.5	161	55.0
ICPC2 code missing	97	13.4	47	16.0
**Visit to primary health care facility**	958	8.3	286	2.6
Visit duration: median 15 min [IQR 0–20] (0–24 h) and 20 min [IQR 0–30] (24–48 h)				
Primary health care visit associated with initial non-conveyance mission				
ICPC2 code mapped to ICD10 code	95	9.9	25	8.8
ICPC2 code does not map to ICD10 code	785	81.7	237	83.2
ICPC2 code mapped to ICD10 category	154	16.0	41	14.4
ICPC2 code does not map to ICD10 category	726	75.5	221	77.5
ICPC2 code or ICD10 code missing	81	8.4	23	8.1
**Visit to ED**	519	4.4	86	0.8
Visit duration: 65% of visits less than 1 day				
ED visit associated with initial non-conveyance mission				
ICPC2 code mapped to ICD10 code	63	12.1	10	11.8
ICPC2 code does not map to ICD10 code	376	72.3	67	78.8
ICPC2 code mapped to ICD10 category	85	16.3	17	20.0
ICPC2 code does not map to ICD10 category	354	68.1	60	70.6
ICPC2 code or ICD10 code missing	81	15.6	8	9.4
**Hospitalization**	184	1.6	30	0.3
Visit duration: median 2 days [IQR 1–4.5] (0–24 h) and 1 day [IQR 1–2] (24–48 h)				
Intensive care	32	0.3	3	0.03
High dependency unit	62	0.5	12	0.1
Hospitalization associated with initial non-conveyance mission				
ICPC2 code mapped to ICD10 code	6	3.3	5	16.7
ICPC2 code does not map to ICD10 code	178	96.7	25	83.3
ICPC2 code mapped to ICD10 category	16	8.7	7	23.3
ICPC2 code does not map to ICD10 category	168	91.3	23	76.6
ICPC2 code or ICD10 code missing	29	15.8	1	3.3

Predictors of adverse events are shown in Table 7. EMS arrival time at night and older patient age were common predictors of many events. Furthermore, EMS re-contact (0–24 h) was more likely when the patient had refused conveyance than when the EMS had treated the patient at the scene or there was no need for conveyance (aOR 1.79; 95% CI 1.37 to 2.34). The usage of alcohol (aOR 1.37; 95% CI 1.10 to 1.71) was also associated with EMS re-contact (0–24 h). Non-urgent mission (aOR 1.49; 95% CI 1.25 to 1.77), ALS unit attendance (aOR 1.43; 95% CI 1.16 to 1.77), and rural area (aOR 1.74; 95% CI 1.51 to 1.99) also increased the likelihood of subsequent visits to a primary health care facility. If the patient was handed over to the police, the likelihood of an ED visit (0–24 h) increased (aOR 2.16; 95% CI 1.34 to 3.49).

**Table 7:** Logistic regression model of EMS re-contact, primary health care facility/ED attendance, and hospitalization

Odds Ratio Estimates	EMS re-contact 0–24 h	EMS re-contact 24–48 h	Visit to primary health care 0–24 h	Visit to primary health care 24–48 h	Visit to ED 0–24 h	Visit to ED 24–48 h	Hospitalization 0–24 h
	Adjusted OR (95%)	Adjusted OR (95%)	Adjusted OR (95%)	Adjusted OR (95%)	Adjusted OR (95%)	Adjusted OR (95%)	Adjusted OR (95%)
**Mission priority**							
**CD vs. AB**	0.975 (0.814–1.168)	1.249 (0.930–1.676)	1.488 (1.254–1.767)	1.585 (1.167–2.155)	1.258 (1.007–1.571)	0.935 (0.570–1.534)	1.214 (0.752–1.961)
**EMS arrival time**							
**20:00–8:00 vs. 8:00–20:00**	1.180 (1.007–1.383)	0.913 (0.714–1.168)	1.818 (1.579–2.094)	1.113 (0.872–1.420)	1.530 (1.269–1.845)	1.068 (0.685–1.664)	1.235 (0.838–1.819)
**EMS units**							
**ALS vs. BLS**	0.933 (0.759–1.147)	0.972 (0.709–1.333)	1.431 (1.155–1.773)	1.123 (0.805–1.566)	0.612 (0.489–0.766)	0.737 (0.423–1.284)	1.132 (0.712–1.799)
**Rural vs. urban**	0.835 (0.709–0.983)	1.011 (0.790–1.293)	1.735 (1.511–1.993)	1.497 (1.175–1.907)	1.008 (0.834–1.219)	1.346 (0.866–2.090)	1.188 (0.802–1.761)
**Age, years**							
**< 15 vs. 15–64**	0.241 (0.099–0.590)	0.341 (0.107–1.086)	0.545 (0.341–0.871)	0.618 (0.284–1.347)	0.898 (0.552–1.459)	0.963 (0.289–3.211)	0.429 (0.119–1.543)
**65–84 vs. 15–64**	1.594 (1.326–1.917)	1.561 (1.177–2.071)	1.411 (1.203–1.656)	1.339 (1.015–1.765)	0.836 (0.676–1.033)	1.039 (0.616–1.753)	1.442 (0.918–2.267)
**> 85 vs. 15–64**	1.712 (1.354–2.165)	1.507 (1.045–2.173)	1.147 (0.931–1.412)	0.994 (0.686–1.439)	0.828 (0.626–1.095)	1.427 (0.772–2.639)	2.139 (1.204–3.799)
**Alcohol**	1.370 (1.100–1.707)	1.376 (0.977–1.938)	0.373 (0.284–0.489)	0.433 (0.273–0.685)	0.567 (0.422–0.760)	0.622 (0.299–1.290)	0.723 (0.381–1.374)
**Reason for non-conveyance**							
**Patient was handed over to police vs. treatment at scene**	1.086 (0.665–1.773)	0.836 (0.359–1.948)	0.539 (0.262–1.106)	0.211 (0.029–1.527)	2.161 (1.338–3.489)	2.157 (0.632–7.358)	0.773 (0.268–2.223)
**Patient refused conveyance vs. treatment at scen**	1.792 (1.370–2.344)	1.324 (0.837–2.093)	0.776 (0.544–1.107)	1.100 (0.653–1.853)	1.292 (0.899–1.856)	1.669 (0.746–3.733)	2.190 (1.073–4.471)
	Univariate OR (95%)	Univariate OR (95%)	Univariate OR (95%)	Univariate OR (95%)	Univariate OR (95%)	Univariate OR (95%)	Univariate OR (95%)
**Mission Priority**							
**Non-urgent vs. urgent**	1.089 (0.915–1.295)	1.367 (1.028–1.818)	1.493 (1.267–1.760)	1.587 (1.177–2.141)	1.217 (0.987–1.500)	0.981 (0.607–1.583)	1.402 (0.899–2.186)
**EMS arrival time**							
**20:00–8:00 vs. 8:00–20:00**	1.160 (0.998–1.349)	0.951 (0.753–1.201)	1.748 (1.528–1.999)	1.079 (0.852–1.365)	1.433 (1.201–1.710)	0.984 (0.642–1.507)	1.199 (0.834–1.723)
**EMS units**							
**ALS vs. BLS**	0.895 (0.736–1.089)	0.877 (0.649–1.184)	1.574 (1.285–1.928)	1.139 (0.825–1.572)	0.668 (0.542–0.824)	0.717 (0.430–1.196)	1.094 (0.713–1.680)
**Rural vs. urban**	0.874 (0.746–1.024)	0.952 (0.751–1.208)	1.763 (1.542–2.016)	1.541 (1.217–1.951)	0.928 (0.770–1.117)	1.229 (0.835–1.990)	1.249 (0.857–1.821)
**Age, years**							
**< 15 vs. 15–64**	0.191 (0.079–0.466)	0.299 (0.095–0.947)	0.720 (0.462–1.121)	0.745 (0.344–1.614)	0.894 (0.561–1.425)	0.947 (0.290–3.094)	0.420 (0.119–1.478)
**65–84 vs. 15–64**	1.381 (1.166–1.635)	1.535 (1.182–1.994)	1.681 (1.449–1.953)	1.645 (1.265–2.139)	0.851 (0.698–1.036)	1.000 (0.611–1.637)	1.587 (1.056–2.384)
**> 85 vs. 15–64**	1.425 (1.154–1.760)	1.439 (1.032–2.007)	1.363 (1.121–1.657)	1.209 (0.846–1.727)	0.848 (0.654–1.100)	1.440 (0.820–2.532)	2.206 (1.310–3.715)
**Alcohol**	1.307 (1.080–1.581)	1.157 (0.854–1.569)	0.372 (0.290–0.479)	0.768 (0.591–0.998)	0.682 (0.352–1.321)	0.682 (0.352–1.321)	0.689 (0.391–1.211)
**Reason for non-conveyance**							
**Patient was handed over to police vs. treatment at scene**	1.065 (0.665–1.707)	0.804 (0.355–1.822)	0.357 (0.189–0.672)	0.121 (0.017–0.866)	1.851 (1.198–2.861)	1.455 (0.456–4.642)	0.657 (0.254–1.699)
**Patient refused conveyance vs. treatment at scene**	1.794 (1.390–2.315)	1.293 (0.831–2.012)	0.537 (0.380–0.760)	0.834 (0.501–1.390)	1.205 (0.854–1.699)	1.374 (0.631–2.992)	1.763 (0.899–3.455)

In addition, the univariate analyses showed that high NEWS2 score (score of 7 vs. 3 in any individual parameter: OR 3.16, 95% CI 1.54 to 6.48; 7 vs. 0–4: OR 3.44, 95% CI 1.78 to 6.67) or a 1-point increase in the NEWS2 score (OR 1.09; 95% CI 1.04 to 1.15) increased the likelihood of EMS re-contact (0–24 h). There were also significant associations between BLS unit attendance and subsequent ED visit in 0–24 h (BLS vs. ALS units: OR 1.50; 95% CI 1.21 to 1.85). Longer distance to primary health care or ED predicted subsequent visits to a primary health care facility (0–24 h > 40 km vs. 21–40 km: OR 1.30, 95% CI 1.05 to 1.61; > 40 km vs. 5–20 km: OR 2.26, 95% CI 1.83 to 2.79; > 40 km vs. < 5 km: OR 1.82, 95% CI 1.48 to 2.24; 24–48 h > 40 km vs. 21–40 km: OR 1.54, 95% CI 1.08 to 2.21; > 40 km vs. 5–20 km: OR 2.42, 95 CI 1.69 to 3.45; > 40 km vs. < 5 km: OR 2.14, 95% CI 1.51 to 3.03). In addition, non-specific reasons for care increased the probability of EMS re-attendance in 24–48 h (OR 1.304; 95% CI 1.00 to 1.70). These predictors based on univariate analyses did not show any significant associations with other outcomes. Gender (*p*-value ≥0.054) and if there was less than an hour to complete a shift (p = ≥0.094), did not predict any outcomes in this study.

**Fig. 3:**
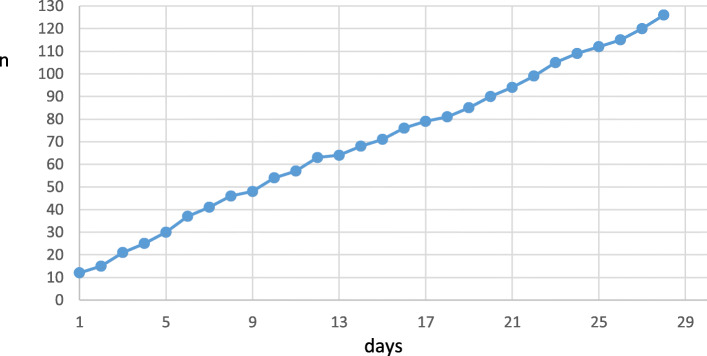
Deceased patients

After exclusion of patients for whom an end of life decision had been made either before or during EMS attendance via telephone consultation with EMS physician (*n* = 55, median age 85 years), there were 126 patients who died within 28 days (Fig. 3). In this group, the mission leading to the non-conveyance decision occurred most often at home (61%, *n* = 77). The remaining missions occurred at health care or social service units (37%, *n* = 47) and public places (2%, *n* = 2). Among the deceased patients, the place of death was at home in 17% (*n* = 22), at health care or social service units in 81% (*n* = 102), and in public places in 2% (n = 2). Overall, 10 patients refused conveyance to the ED. The median age of the deceased patients was 83 years. Emergency physicians or primary care physicians were consulted in 51% of the cases. Based on clinical re-evaluation by JP, TI, and JK, 25% (*n* = 32) of the deaths were related to the initial non-conveyance mission. In four cases (0.03% of the initial non-conveyance missions), the non-conveyance decision was not appropriate according to the clinical re-evaluation. In the first case, the patient with symptoms of gastroesophageal reflux had ST-elevation myocardial infarction but a 12-lead ECG had not been recorded. In the three remaining cases, the patients had shortness of breath and swelling of the foot due to coronary disease leading to heart failure, non-specific symptoms due to pneumonia, and aortic dissection with typical back pain. In two of these four cases, a physician had been consulted. In addition, one patient who refused treatment and later died would have clearly benefited from conveyance to the ED.

## Discussion

The main findings of this study were as follows. Firstly, 4 in 5 (83.9% in 0–24 h) of non-conveyed patients had no adverse events after the non-conveyance mission. Secondly, patients were mainly in good condition; the NEWS2 scores were low and duration of visits to primary health care or EDs short. The reasons for the adverse events seem to be different than the reason for the initial EMS mission. Thirdly, 0.03% of the non-conveyance decisions seem to be related to a patient’s death, where re-evaluation showed poor clinical judgement and/or clinical treatment protocol violation.

From the perspective of patient safety, it is important that the majority of non-conveyed patients did not have any subsequent events during the follow-up period, which is in line with previous studies [21, 31]. We found that EMS re-contacts, visits to primary health care or ED and hospitalization were relatively rare after non-conveyance missions. Two previous reviews indicate that there are many studies focusing on specific populations in which the sample size is small or the follow-up is by telephone, which may lead to bias because the follow-up of a large number of patients may fail [5, 17]. Thus, comparisons with our findings are challenging. However, the subsequent event rates in this study were roughly the same as in other similar studies [21, 31].

Our study indicates that the 24-h period after an EMS visit seems to be critical. The highest incidence of all outcomes occurred within 0–24 h after the initial non-conveyance mission, which was noted previously [21]. A longer follow-up period could have provided more information, but the likelihood of an adverse event being due to some other reason would have increased.

Our study shows that, in the case of EMS re-contact, primary health care or ED attendance, and hospitalization, the patients were mainly in good condition. The new mission (EMS re-contact) was commonly non-urgent, the patients’ NEWS2 scores were low, and almost half of these cases ended in a new non-conveyance decision. The visits to primary health care facilities or EDs were mostly short. On the other hand, 32 patients (0.3%) needed intensive care and 62 patients (0.5%) were treated in high-dependency units after the initial non-conveyance case. These findings are similar to a Swedish study [10]. However, it is difficult to assess whether these patients’ critical condition was derived from the EMS’s poor clinical judgement and incorrect non-conveyance decision. When comparing the preliminary diagnosis (adjusted ICPC2) to new ICPC2 codes in case of EMS re-contact or discharge diagnoses (ICD10) from primary health care, ED, or after hospitalization, these adverse events were usually not related to the initial non-conveyance mission. In addition, we found that there were many subsequent visits to primary health care, which may be an indication of impaired access to primary health care, especially in the evening and at night.

Our logistic regression model (Table 7) and previous studies [21, 32] indicate that older age is a risk factor for adverse outcomes. This may be due to elderly patients’ complex symptoms and many comorbidities and medications. We also found that patients’ refusal of conveyance and the use of alcohol predicted an EMS re-contact in 0–24 h. One explanation may be that these patients do not know how to handle their problems and an EMS re-call is the easiest choice. Other studies have reported that the refusal is associated with ED visits [33] and alcohol increases the likelihood of non-conveyance [3].

Decision-making at night is challenging [34]. Our results show that EMS arrival at night increases the likelihood of 3 in 4 primary outcomes of this study. However, the end of a work shift was not associated with re-contacts, which may indicate that EMS providers consider the patient’s needs even though the shift is close to its end. However, EMS arrival at night, non-urgent mission, ALS unit, rural area, and longer distance to a health care facility or ED were related to subsequent visits to primary health care. There is a possibility that this demonstrates appropriate use of health care resources to avoid unnecessary conveyance to the ED. Geographic variation in the EMS context is high [35], but the impact of geography on a patient’s outcome is unclear. However, the on-scene time is reported to be high in rural areas [36] and in the case of non-conveyance [3]. Understandably, visits to primary health care were related to non-urgent missions. In contrast, it seems that EMSs can safely discharge urgent missions such as hypoglycemia and other chronic diseases at the scene after appropriate assessment and treatment. Previous studies have shown that non-conveyance is challenging and requires competence [5, 34]. Indeed, there are a number of factors that are related to non-conveyance decisions [3, 8, 37–39]. Our study demonstrated that ALS units are associated with re-contact in primary health care. This may be due to the longer education of EMS care providers in ALS units, which may be associated with more appropriate decision-making related to the use of primary health care and ED resources. On the other hand, based on the univariate analyses, BLS units increased the risk of subsequent ED visits. This raises the question of whether these visits are related to the BLS units’ competence. However, more studies are needed.

Moreover, an Australian study indicated that, when the EMS discharged patients at the scene, there was an increased risk of adverse events compared to patients discharged from the ED [21]. Notably, subsequent contacts with health care do not automatically mean that patient safety is compromised [5, 30].

Abnormal vital signs have been found as a common predictor of adverse events after EMS non-conveyance [21]. We found that, if the patient’s NEWS2 score increases by 1 point or the score is high (> 7 points), the risk of EMS re-contact increases. Non-specific complaints lead to a number of adverse outcomes in both the EMS context and EDs [19, 20, 40]. Surprisingly, we did not find similar results. Based on univariate analyses, non-specific complaints were only related to EMS re-contact in 24–48 h.

### Limitations

This study has several limitations, three of which were described previously (excluded patients, the use of adjusted ICPC2 classification, and NEWS2 score calculation) [3].

The register of ED visits included the date, but the exact visit time was mainly missing. Thus, the initial non-conveyance case was judged to have occurred first and the 0–24 h ED visit to have occurred on the same day or the following day. Furthermore, the register of visits to primary health care includes chronic disease monitoring. Therefore, the rate of visits to primary health care and EDs for real adverse events after the non-conveyance mission may be lower. On the other hand, some patients may have sought further care at private clinics, but all patients with severe or even moderate deterioration would have been sent to an ED.

The reasons for care (adjusted ICPC2) were compared to the discharge diagnoses (ICD10) from the primary health care facilities or EDs or to diagnoses after hospitalization in order to determine whether these events were related to the initial non-conveyance mission. Notably, the ICPC2 chosen by EMSs is based on symptoms and signs present at the time of the EMS contact. The time between ICPC2 and discharge diagnosis, further examination, and the treatment given may affect the discharge diagnosis. Thus, the real rate of adverse events due to the same reason as the initial non-conveyance case may be higher.

Only unexpected deaths were analyzed, and therefore patients with end of life decisions were excluded. However, it is possible that this approach did not identify all of these patients due to missing information. When assessing the risk factors for adverse events, 28-day mortality and hospitalization in 24–48 h were excluded from our multivariable logistic regression model as dependent variables because these events were very rare in our data set.

In Finland, EMSs and EDs are encouraged to plan emergency patient pathways together. Therefore, triaging and assessing the need for conveyance and non-conveyance decisions are commonly made by the EMS. This practice and the level and scope of education differs between countries; therefore, the generalizability of the results may be limited [23].

## Conclusion

Most non-conveyance patients did not have adverse events in the follow-up period. In the case of EMS re-contact or visits to a primary health care facility or ED and hospitalization, the patients were mainly in good condition and the reason was often something other than the initial non-conveyance mission. A small proportion of non-conveyed patients were later in critical condition; deaths were very infrequent. From a patient safety perspective, the subsequent 24 h after a non-conveyance decision are critical. EMS non-conveyance seems to be a safe and rational use of limited resources and, therefore, is a solution that reduces unnecessary patient conveyance to EDs.

## Data Availability

The data of this study is not available due to patients’ privacy.

## Abbreviations

EMS: Emergency medical services
ED: Emergency Department
ALS: Advanced Life Support
BLS: Basic Life Support
NEWS2: National Early Warning Score
